# Verruculogen associated with *Aspergillus fumigatus *hyphae and conidia modifies the electrophysiological properties of human nasal epithelial cells

**DOI:** 10.1186/1471-2180-7-5

**Published:** 2007-01-23

**Authors:** Khaled Khoufache, Olivier Puel, Nicolas Loiseau, Marcel Delaforge, Danièle Rivollet, André Coste, Catherine Cordonnier, Estelle Escudier, Françoise Botterel, Stéphane Bretagne

**Affiliations:** 1 Laboratoire de Parasitologie-Mycologie, Hôpital Henri Mondor (AP-HP), Université Paris 12, and UMR BIPAR 956, Créteil, France; 2 Laboratoire de Pharmacologie-Toxicologie, Institut National de Recherche Agronomique, Toulouse, France; 3 CNRS URA 2096, CEA Saclay DSV/DBJC/SBFM, Gif sur Yvette, France; 4 Inserm U 492, Université Paris 12, Créteil, France; 5 Service d'ORL et de Chirurgie Cervicofaciale, Hôpital Henri Mondor (AP-HP) and Hôpital Intercommunal de Créteil, France; 6 Service d'Hématologie clinique, Hôpital Henri Mondor (AP-HP), Créteil, France; 7 Département de Génétique, Cytogénétique et Embryologie, Groupe Hospitalier Pitié-Salpêtrière (AP-HP), Paris, France

## Abstract

**Background:**

The role of *Aspergillus fumigatus *mycotoxins in the colonization of the respiratory tract by conidia has not been studied extensively, even though patients at risk from invasive aspergillosis frequently exhibit respiratory epithelium damage. In a previous study, we found that filtrates of *A. fumigatus *cultures can specifically alter the electrophysiological properties of human nasal epithelial cells (HNEC) compared to those of non pathogenic moulds.

**Results:**

We fractionated the organic phase of filtrate from 3-day old *A. fumigatus *cultures using high-performance liquid chromatography. The different fractions were tested for their ability to modify the electrophysiological properties of HNEC in an *in vitro *primary culture model.

The fraction collected between 20 and 30 min mimicked the effects of the whole filtrate, i.e. decrease of transepithelial resistance and increase of potential differences, and contained secondary metabolites such as helvolic acid, fumagillin, and verruculogen. Only verruculogen (10^-8 ^M) had effects similar to the whole filtrate. We verified that verruculogen was produced by a collection of 67 human, animal, plant and environmental *A. fumigatus *isolates. Using MS-MS analysis, we found that verruculogen was associated with both mycelium and conidia extracts.

**Conclusion:**

Verruculogen is a secondary metabolite that modifies the electrophysiological properties of HNEC. The role of these modifications in the colonization and invasion of the respiratory epithelium by *A. fumigatus *on first contact with the epithelium remains to be determined.

## Background

*Aspergillus fumigatus *is associated with many human health conditions [1]. It is the most common cause of invasive aspergillosis, an opportunistic infection whose incidence increases in immunocompromised patients [2]. Aspergillosis is also prevalent in animals, such as dogs and birds [3,4]. *A. fumigatus *is responsible for more human and animal diseases than other moulds even though their conidia are usually outnumbered by spores of other mould species in inhaled air, suggesting that *A. fumigatus *has special properties that enable it to colonize the lungs of its hosts [5]. Most studies with *A. fumigatus *have focused on the interactions of the fungus with alveolar macrophages [6,7] or with cell line A549 from pneumocytes [8,9]. However, the first tissue usually encountered by the inhaled conidia is the airway epithelium, and it is frequently damaged in patients at risk for invasive aspergillosis [10]. We previously studied the interactions between *A. fumigatus *and the respiratory epithelium using human nasal epithelium cell (HNEC) cultures [11,12]. After one week of culture, the HNEC are organized in a pseudostratified epithelium with mucus and ciliated cells, very similar to the in vivo airway epithelium [12].

We found that a filtrate from *A. fumigatus *cultures has specific electrophysiological effects on HNEC compared with one from fungi not responsible for invasive infection [11]. Effects included a decrease in transepithelial electrical resistance and polarization of the epithelium. These changes to the epithelium properties could play a role in the ability of *A. fumigatus *to colonize the respiratory tract. In the present study, in order to identify which compounds were responsible for these changes, we used high-performance liquid chromatography with diode-array detection (HPLC-DAD) to fractionate the filtrate of *A. fumigatus *culture. Thus, we identified verruculogen as the toxin responsible for the toxic effects observed in the epithelial cell model. To determine whether verruculogen was associated with conidia upon its first contact with epithelial cells, the presence of verruculogen was specifically monitored in conidial extracts by mass spectrometry analysis. Moreover, since every isolate of *A. fumigatus *is potentially responsible for invasive aspergillosis (as previously shown using polymorphic microsatellite markers [13,14]), the ability of every isolate to produce verruculogen was a prerequisite for involving verruculogen as a pathogenic factor. The production of verruculogen was therefore verified in a collection of 67 human, animal, plant and environmental *A. fumigatus *isolates.

## Results

**Figure 1:**
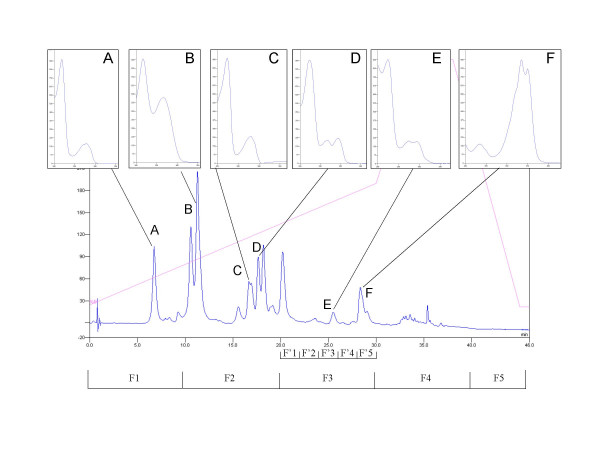
HPLC – Chromatogram at 270 nm of *A. fumigatus *IP 2279-94, growth at 25°C in MEM and UV spectra of metabolites detected in this culture: fumigaclavine A [A], gliotoxin [B], fumigaclavine C [C], fumitremorgin C [D], verruculogen [E], and fumagillin [F]. The first fractionation was performed as follows: four 10-minute fractions (F1-4) and one 6-minute fraction (F5). F3 fraction was secondarily split into two-minute fractions (F'1-5). Pink line: gradient line.

We first fractionated the metabolites and analyzed the organic phase profile obtained with 25°C fungi cultures, to obtain enough metabolites to be seen in HPLC analysis. For 25°C fungi cultures, we detected and identified fumigaclavine A and C, fumitremorgin C, verruculogen, and fumagillin by comparison with authentic standards (Figure 1). For 37°C culture, other metabolites were identified on the basis of their UV spectrum: fumigaclavine A, gliotoxin, fumiquinazoline F, fumitremorgin C, fumagillin, and helvolic acid. When testing the different HPLC fractions and commercial products, the results obtained after 30 min or 3 hours of exposure were similar. Therefore, only the 30 min results were retained.

**Figure 2:**
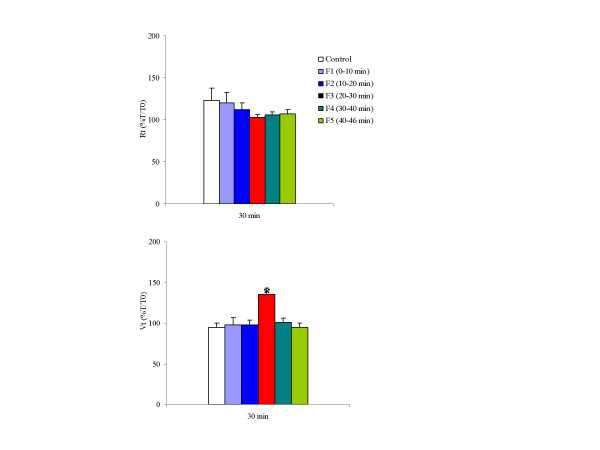
Effect of the five fractions obtained by HPLC-DAD of the organic phase of the 37°C *A. fumigatus *culture filtrate at 1/2 h. Only F3 (20–30 min) showed a significant increase in transepithelial potential differences (Vt) and a decrease, albeit not significant, in transepithelial resistance (Rt).  *: significant difference compared with HNEC culture medium with DMSO as controls (p < 0.05).

**Figure 3:**
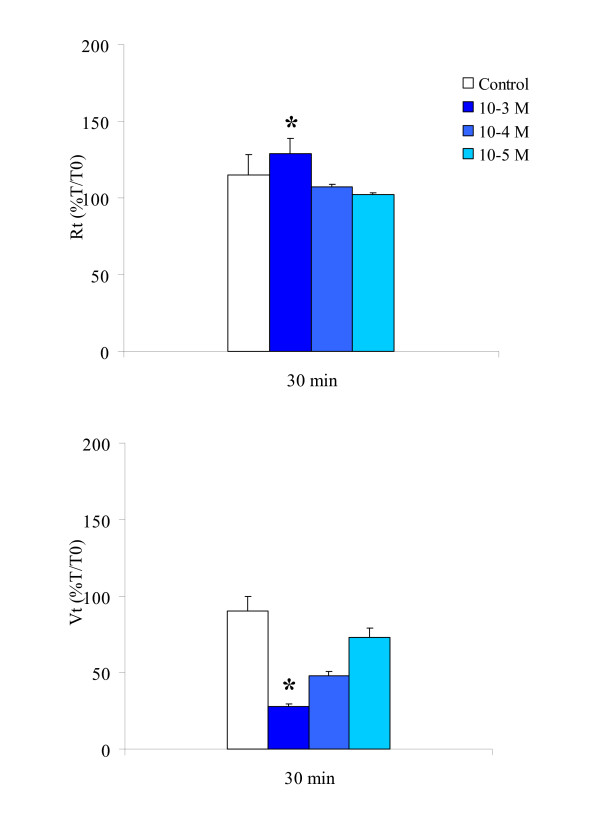
Effect of serial dilutions of standard fumagillin at 1/2 h. The first significant effects observed, i.e. a decrease in transepithelial potential differences (Vt) and an increase in transepithelial resistance (Rt), were the opposite of those observed with the whole organic phase. *: significant difference compared with HNEC culture medium with DMSO as controls (p < 0.05).

**Figure 4:**
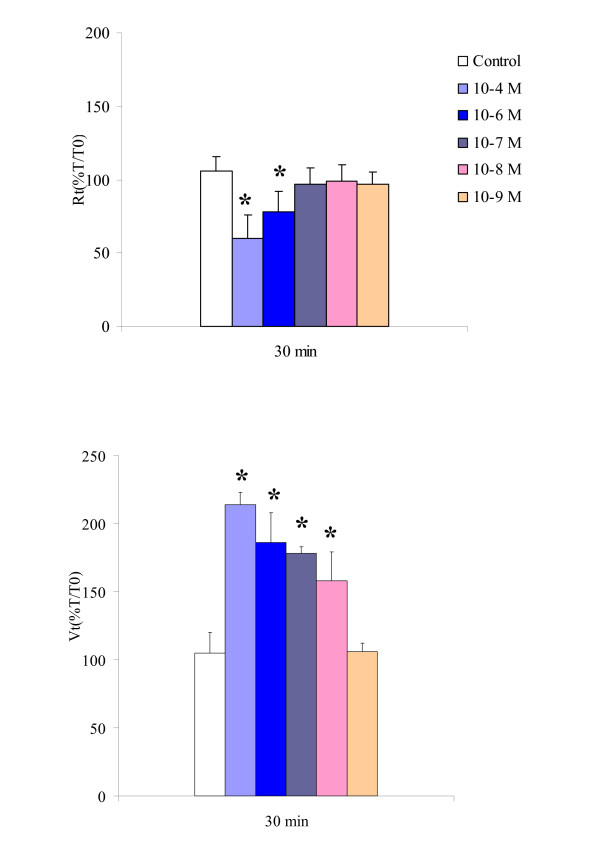
Effect of serial dilutions of standard verruculogen at 1/2 h. The first significant effects observed, i.e. an increase of transepithelial potential differences (Vt) and a decrease of transepithelial resistance (Rt), were similar to those observed with the whole organic phase. *:significant difference compared with HNEC culture medium with DMSO as controls (p < 0.05).

At 37°C, only the 20–30 min fraction 3 (F 3) increased the Vt (Figure 2). As this fraction was known to contain the secondary metabolites helvolic acid, fumagillin and verruculogen, the effects of different concentrations of these commercially available compounds were tested. Helvolic acid had no significant effect (data not shown). The effects of fumagillin are summarized in Figure 3 and were opposite to the effects of F3. Only verruculogen showed effects similar to those of F3. In Figure 4, the effects of different concentrations of verruculogen are presented, ranging from 10^-4 ^to 10^-9 ^M. The effects on Rt and Vt were slightly dissociated, as 10^-4 ^and 10^-6 ^M verruculogen decreased Rt and increased Vt, whereas 10^-7 ^and 10^-8 ^M verruculogen only increased Vt with no significant effect on Rt.

To verify that verruculogen was responsible for the effects of fraction 3, F3 was further fractionated into five two-minute fractions (F'1-5). In these HPLC conditions, verruculogen was recovered in F'3 (24–26 min) while helvolic acid and fumagillin were eluted together in F'5 (28–30 min). Among the five tested fractions, only F'3 displayed results similar to those of F3. Since verruculogen is produced in greater amounts at 25°C than at 37°C [15], we performed similar experiments with the organic phase obtained at 25°C to look for a concentration effect. For 25°C culture, F'3 revealed an increase in Vt and a decrease in Rt within the same range as 10^-6 ^M standard verruculogen, whereas the effects observed with F'3 of 37°C culture were similar to those obtained with lower concentrations of verruculogen (data not shown).

**Figure 5:**
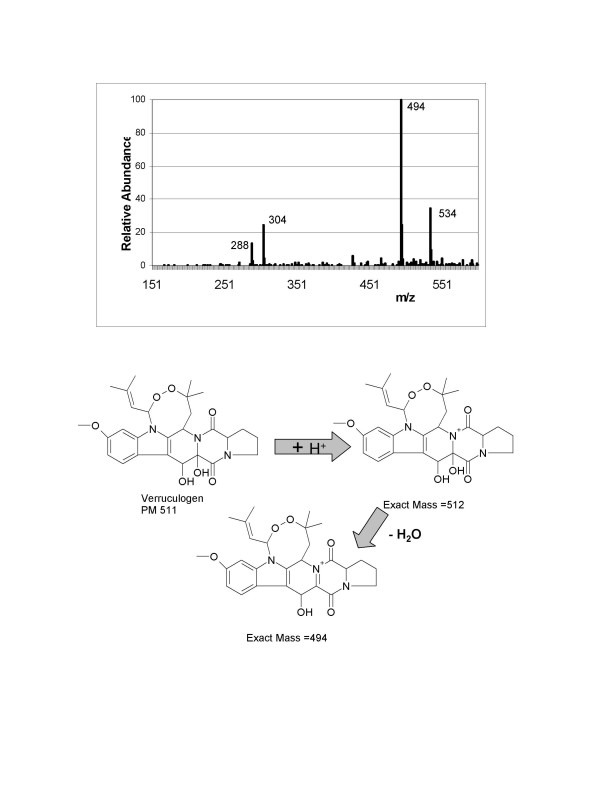
Positive mode electrospray ionization MS analysis of the F'3 fraction. Despite the fact that the molecular ion at m/z 512 (M+ H)^+ ^is not observed, the presence of ions at both m/z 494 (M- H_2_O)^+ ^and m/z 534 (M + Na)^+ ^enables this compound to be identified as verruculogen [16].

Fraction F'3 was analyzed by mass spectrometry and verruculogen was identified on the basis of electron ionization MS and UV spectral analysis, after comparison with a reference standard. In the analysis of fraction F'3, the mass spectrum of verruculogen showed two major ions at *m/z *494 and 534, corresponding to loss of water and sodium adduct, respectively (Figure 5). The collision-induced dissociation MS-MS spectrum of the 494 ion led to fragment at *m/z* 410, identical to that of authentic verruculogen [16].

The amounts of fumagillin and verruculogen were estimated by HPLC-DAD using standard curves. F'3 contained 4.15 μg of fumagillin for 37°C culture and 3.96 μg for 25°C culture. Therefore, the concentration of fumagillin in culture filtrate used to challenge the HNEC was 0.36 10^-6 ^M, more than 300 times lower than the lowest concentration, 1.1 10^-4 ^M, of purified fumagillin resulting in a significant effect (Figure 3), thus definitively excluding fumagillin as the toxin involved in our observations. For verruculogen, F'3 of 37°C culture contained less than 80 ng of verruculogen and Fraction F'3 of 25°C culture contained 2.1 μg of verruculogen. The concentrations obtained after resolubilization in DMSO and dilution to challenge HNEC corresponded to 0.6 10^-6 ^M for 25°C culture and 1.4 10^-8 ^M of verruculogen for 37°C culture. Compared with the results obtained with the serial dilutions of standard verruculogen (Figure 4), fraction F'3 of 25°C culture showed an increase in Vt and Rt, as did the 10^-6 ^M concentration, whereas F'3 of 37°C culture presented a decrease in Vt in the same way as the 10^-8 ^concentration.

Verruculogen was detected by HPLC-DAD in the 67 *A. fumigatus *hyphal and conidial extracts tested, from 0.13 to 17.2 μg/g of wheat. One extract of conidial fractions was also analyzed by mass spectrometry to verify the presence of verruculogen. In positive mode, verruculogen (molecular weight: 511 g/mol) was detected in conidial extract. The presence of verruculogen was confirmed after 14.8 min of elution, at which time its mass spectrum corresponded to that of standard verruculogen. In these extracts, seven other compounds were also specifically detected (emodin, gliotoxin, fumigaclavine C and four compounds of the tremorgen family, namely tryprostatin A, TR2, fumitremorgin C, and dihydroxy-fumitremorgin C).

## Discussion

The production of toxins by *A. fumigatus *may help the fungus to colonize and invade the respiratory epithelium by modifying the natural clearance of the respiratory tract. Previous research has shown that *A. fumigatus *culture filtrate modifies the transepithelial resistance (Rt) and transepithelial potential differences (Vt) of HNEC, an *in vitro *model of the air-liquid interface of airway epithelium [11]. The aim of this study was to use HPLC and MS-MS to identify which toxins produced by *A. fumigatus *are responsible for these modifications. Our data suggest that verruculogen, which has never been implicated in invasive aspergillosis, is one of the probable candidates.

The fact that *A. fumigatus *produces a number of biologically active substances that slow ciliary beating, damage epithelium, and that may affect colonization of the airways has already been reported using culture explants [17]. Among these substances, such toxins as gliotoxin, fumagillin, and helvolic acid have been implicated in the pathogenesis of aspergillosis [18]. Our results did not suggest the involvement of any of these toxins in the effects observed on our in vitro model of respiratory epithelium. The HPLC fraction known to contain gliotoxin had no detectable effect, suggesting that the concentration of gliotoxin in the fungal filtrate was too low to play a role in the observed effects. For fumagillin, the effects were different from those of the whole *A. fumigatus *culture filtrate and were only observed with concentrations above those found in the culture filtrate. With the helvolic acid, we did not observe any effects at the tested concentrations based on the residual extract HPLC analysis. Therefore, we concluded that none of these metabolites were responsible for the electrophysiological modifications of HNEC. Nevertheless, their role, especially in association, cannot be completely ruled out. For instance, gliotoxin is known to be produced by *A. fumigatus *during the exponential growth phase at 37°C [19]. Therefore, the role of gliotoxin may be minimal in the first days of colonization, after the seeding of airway epithelium, but become crucial at a later stage of infection. Gliotoxin has been detected in patients suffering from aspergillosis [20] and in bovine udder infected with *A. fumigatus *[21].

Our results strongly suggest that verruculogen is one of the candidate toxins, if not the sole toxin, responsible for the changes of HNEC observed with culture filtrate. Our arguments are based on the fact that: (i) only the fraction containing verruculogen modified Vt and Rt, (ii) the concentration of verruculogen in the organic extract corresponds to the concentration obtained from the standard curve produced with pure commercial toxin, (iii) the temperature of 25°C, which fosters the production of verruculogen by *A. fumigatus*, led to increased effects, and that (iv) heating the filtrate at 100°C for 10 min did not modify the effects on HNEC [11] and is known not to modify the structure of verruculogen (this was verified by HPLC analysis; not shown).

Verruculogen is one of the tremorgenic mycotoxins produced by fungi belonging to the genera *Penicillium *and *Aspergillus *that elicit intermittent or sustained tremors (staggers syndromes) in vertebrate animals [22,23]. The clinical symptoms typically observed during mycotoxin poisoning include diminished activity and immobility, followed by hyper-excitability, convulsions, muscle tremor, ataxia, and tetanic seizures. However, death rarely occurs and these symptoms are reversible if the animals are removed from the toxic-feed source. The concentrations detected in our *A. fumigatus *filtrates are considerably lower than those reported when clinical symptoms of poisoning are observed in ruminants. No data are yet available in relation to the concentration dependence of human susceptibility. Verruculogen is known as an inhibitor of K+ channels in synaptic in vitro models [23,24]. Since verruculogen has never been tested on HNEC, additional research is needed to identify the prime action site on HNEC. The action of verruculogen could be extended to other targets such as DNA [25].

In order to include verruculogen as one of the virulence factors of *A. fumigatus*, toxin should be present in all the *A. fumigatus *isolates. No specific isolates have been shown to be more pathogenic than others in humans [13,26,27] or birds [3]. According to some authors, 40% to 91% of the *A. fumigatus *isolates produce verruculogen [28,29]. The production of mycotoxins at an easily detectable level is very dependent on culture medium and temperature. For instance, verruculogen is produced in larger amounts at 25°C than at 37°C [15]. Generally, greater amounts of mycotoxins are detected in grain cultures than in standard liquid cultures. Moreover, the production of toxins on grains is more physiological for a mould than the production of toxins in microbiological medium such as PDA or Sabouraud medium. Using autoclaved grains as a culture support, we were able to show that 67 different *A. fumigatus *isolates are able to produce verruculogen. As we do not observe direct cell lysis with low concentrations of verruculogen, further studies are required to test whether verruculogen is a virulent factor in the strictest sense, i.e. a component that damages the host cells [30]. However, a verruculogen-deficient mutant strain would probably not be informative, since the pathogenicity of *A. fumigatus *is multifactorial. Indeed, all experiments performed to prove the role of a specific molecule in the virulence of *A. fumigatus *have failed, with the exception of melanin-deficient mutants [5] and mutants deficient in the siderophore biosynthesis [31,32].

To take on a primary role in the colonization and/or invasion of airway epithelium, verruculogen not only has to be present in every *A. fumigatus *isolate, but also has to be associated with conidia and delivered directly onto the epithelial cells. The presence of verruculogen at the cellular level could alter pulmonary clearance and thereby increase colonization and the risk of invasive aspergillosis. Some authors have found verruculogen in very few conidial extracts [33]. Using MS/MS, we found that verruculogen is associated with conidia (although contamination with residual hyphae is always possible). Therefore, verruculogen can act on its first contact with epithelial cells. Furthermore, the detection of verruculogen in conidial extracts presents opportunities for investigation into the potential role of inhalation in the intake of mycotoxins by humans. The recent detection of abundant respirable ergot alkaloids associated with *A. fumigatus *conidia raises the possibility of the respiratory epithelium as an alternative to the digestive route for the intake of these mycotoxins [34].

## Conclusion

The HPLC-MS and HPLC-MSn analysis techniques used in this study according to recent FDA recommendations [35] confirm the existence of verruculogen and other mycotoxins associated with conidia. The identification of the toxins secreted by *A. fumigatus *and their role in humans is a long-running enterprise involving microbiology, chemistry, and mammalian cell cultures. For the first time, we are including verruculogen as a toxin with deleterious effects on respiratory epithelial cells. Whatever the exact role of verruculogen, it is not the only factor involved in the prevalence of *A. fumigatus *in invasive disease compared with other moulds. Verruculogen is, for instance, secreted by *Penicillium verruculosum*, which has never been implicated in invasive disease. Verruculogen may act first, leading to settlement of the fungus and subsequently allowing other toxins to be produced if the fungus grows. Additionally, verruculogen might be synergistic or antagonistic with other molecules or proteases produced by *A. fumigatus*.

## Methods

### HNEC primary cultures

The HNEC culture conditions were adapted from a culture model originally developed with human tracheobronchial cells [36,37]. Nasal polyps were sampled in patients with nasal polyposis during ethmoidectomy. HNEC cultures were prepared as previously described [11]. In brief, each polyp was placed overnight at 4°C in PBS-antibiotics (100 U/ml penicillin, 100 mg/ml streptomycin, 2.5 μg/ml amphotericin B and 100 mg/ml gentamicin) solution containing 0.1% pronase (Sigma-Aldrich, L'Isle d'Abbeau Chesnes, France). After rinsing, the cells were dissociated in a 0.25% trypsin solution (Invitrogen, Cergy Pontoise, France) and 10^6 ^cells were plated in 12-mm insert wells (Corning BV, Schiphol-Rijk, The Netherlands) with microporous membranes coated with type IV collagen (Sigma-Aldrich) and incubated at 37°C in 5% CO_2_. After 24 h, the culture medium was removed from the apical compartment to obtain an air-liquid interface. The basal compartment was filled with 1 ml of the HNEC culture medium, consisting of DMEM/F12 with 2% Ultroser G (Sigma-Aldrich) with antibiotics (100 U/ml penicillin, 100 mg/ml streptomycin, and 100 mg/ml gentamicin). The HNEC culture medium was changed daily and the electrophysiological properties of the HNEC were checked twice weekly using a microvoltmeter with concentric electrodes (EVOM^® ^World Precision Instruments, Stevenage, UK).

### Fungal strain culture conditions and mycelium mycotoxin extraction

We used *Aspergillus fumigatus *IP 2279-94, originally isolated from a patient with invasive aspergillosis. The strain was cultured on Potato Dextrose Agar (PDA) medium (Sigma-Aldrich) at 37°C for 7 days in the dark. Conidia were collected by gently scraping with spatula and rinsing the PDA Petri dish surface with tryptone salt solution (tryptone 0.1%, 0.8% sodium chloride, 0.1% Tween 80). The suspension was filtered through sterile compresses to remove hyphae. Two hundred and fifty μl of conidial suspensions (2.10^5 ^conidia/ml) were used to inoculate three 500 ml Erlenmeyer flasks each containing 100 ml minimal Eagle's medium (Sigma-Aldrich) supplemented with 5% sterile fetal calf serum, and flasks were then placed in a shaking incubator at 180 rpm for 3 days at 25°C and 37°C, as previously described [38]. After filtration of collected medium through Whatman N°1, the pH of the filtrate was checked and the fungal cultures were extracted with 70 ml chloroform for 4 days at room temperature. The organic phase was evaporated under vacuum at 50°C using a rotary evaporator. The residue was taken up in 200 μl methanol; this suspension was then filtered through a disposable filter with 0.45 μm pores before injection (20 μl) into the chromatograph apparatus.

### Chemicals

Verruculogen, fumagillin, and helvolic acid from Sigma-Aldrich were used without further purification (purities higher than 90%). The sources of the other secondary metabolites used as authentic standards were: P.M. Scott, Health Canada, Ottawa, Canada (fumigaclavine A), J.W. Dorner, National Peanut Research Laboratory, ARS-USDA, Dawson, USA (fumigaclavine C), C. Avendano. Universidad Complutense, Madrid, Spain (fumiquinazoline F), P.G. Mantle, Imperial College, London, UK (TR2) and H. Osada, RIKEN Institute, Wako, Japan (Tryprostatin A). Fumitremorgin C was purchased from Alexis Biochemicals.

### Fractionation of culture filtrates by HPLC and identification of secondary metabolites

The residual extract was fractionated and fungal metabolites were analyzed by HPLC-DAD using a 150 mm × 4 mm Zorbax ODS 5 μm C18 column (Bischoff, Leonberg, Germany) in an HPLC chromatograph apparatus (Kontron, Milan, Italy). HPLC analysis was performed based on a modified Frisvad chromatographic method [39], with a flow rate of 2 ml/min and a linear elution gradient of acetonitrile (solvent B) in 0.2% acetic acid (solvent A). Analysis started with 10% of solvent B, which was increased to 50% after 30 min, and then to 90% four min later, maintained at 90% for a further 4 min, then decreased over 7 min to 10%, and finally maintained at 10% over 1 min. Compounds were identified by comparison with mycotoxin standards analyzed in the same manner. The first fractionation was performed as follows: four 10-minute fractions (F1-4) and one 6-minute fraction (F5) were collected after DAD detection. Fractions were evaporated and taken up in 25 μl DMSO. Ten μl of the DMSO-solubilized HPLC fractions were diluted in 90 μl of HNEC medium, 15 μl of this solution were diluted in 1.5 ml of HNEC medium, and 750 μl of this final solution were used to immerse one HNEC well. After an initial biological activity screening, the positive fraction was split in the same way into two-minute fractions (F'1-5). Fractions were evaporated and taken up as described above.

### Biological activity of the fractions on HNEC

All experiments were performed on 14-day HNEC cultures when cell differentiation was established and stable. All tests were performed in duplicate and each experiment was repeated at least three times with different HNEC cultures. Interactions between HNEC and fungi were evaluated using bioelectrical properties of HNEC cultures, i.e. transepithelial resistances (Rt) and potential differences (Vt), which measure intercellular junction permeability and trans-cellular ionic transport, respectively [40]. For 14-day cultures, the usual range of Rt and Vt is 600 to 800 ohms/cm^2 ^and -20 to -40 mV, respectively. The day before the assay (referred to as T0), the HNEC inserts were immersed in 750 μl of HNEC culture medium. After 30 min to balance electrodes, the Rt and Vt were recorded. After the first measurement, the culture medium was removed from the apical side to recover the air-liquid interface. Twenty-four hours later, 750 μl of the solution containing the tested product were added to the apical side of HNEC cultures and the electrophysiological values were measured at 30 minutes and 3 hours. For a given well, the results were expressed as a percentage variation from the T0 measurements. Calculations were performed using the mean +/- SEM obtained from at least three different cultures.

### Ability of different *A. fumigatus *isolates to produce verruculogen

Sixty-seven *A. fumigatus *strains of the Pharmacology Toxicology Laboratory New Collection (NCPT, INRA Toulouse, France) were examined for their ability to produce verruculogen. These strains were selected from various human, animal, plant and environmental sources and geographical regions. Verruculogen production by *A. fumigatus *strains was measured in autoclaved grains of *Ardente*, a variety of durum wheat. One hundred grams of wheat grains were moistened with 100 ml of sterile distilled water for 4 days at 4°C, before sterilization. Twenty grams of grain were placed in a 140 mm diameter Petri dish. Each dish was inoculated with 250 μl containing 2 × 10^5 ^conidia and incubated at 25°C for 13 days. The fungal cultures were extracted with 100 ml chloroform as described above.

### Mycotoxin extraction from conidia

For detection of mycotoxins associated with conidia, and after having demonstrated that production of verruculogen was effective with every *A. fumigatus *isolate, we pursued experimentation with the NCPT strain 13, which has been used for numerous experiments with mycotoxin production (also available under number NRRL 35693 at the Northern Region Research Laboratory, USDA-ARS, Peoria, Ill, USA). Large amounts of *A. fumigatus *conidia of the NCPT strain 13 were produced on Czapek Yeast Extract Agar (CYA) medium for 14 days at 37°C. Conidia were collected by inverting CYA Petri dishes over a collecting flask. Conidia (10 mg) were gently mixed for 1 min in 2 ml of 0.9% sodium chloride solution containing 0.1% Tween 80. After harvesting, the suspension was filtered through a porosity 2 filter-funnel to remove hyphae and extracted for 4 days by shaking in 10 ml of chloroform at room temperature. The extract was evaporated under vacuum at 50°C on a rotary evaporator. The crude extract was taken up in 500 μl of methanol, and this solution was filtered through a 0.45 μm Teflon filter before liquid chromatography and mass spectrometry analysis.

### HPLC-MS and MS-MS analysis

The LC-MS-MS instrument used was an LCQ DUO Ion Trap from Thermo-Finnigan. The LC separation was done on a 150 × 2.1 mm C18 Kromasil column (Interchim, Montluçon, France) using a 30-minute linear gradient of 10% to 90% acetonitrile in the presence or absence of 0.1% formic acid (presence for positive ionization mode). Electrospray ionization (ESI) was performed at 4.5 kV and the capillary temperature was set to 250°C using a gas flow of 70 ml/min and an auxiliary gas flow of 20 ml/min. MS and MS-MS tuning were performed on 5 μM authentic verruculogen and the MS-MS collision was set at 48%. Whole mycelium and conidial extracts were analyzed in negative and positive ESI mode in full scan and MS-MS mode with an m/z range of 80 to 700. The mass spectra of the positive fraction and verruculogen standard were obtained by infusion in positive ESI mode.

## Authors' contributions

KK, OP, NL, MD, DR, AC, CC, EC, FB, and SB have made substantial contributions to conception and design, acquisition of data, and analysis and interpretation of data. KK and OP have equally contributed to this work. OP, NL, and MD have carried out the mycotoxin analysis. KK, DR, AC, CC, EC, and FB were involved in culture growth, as well as development and biological tests. KK, OP, MD, and SB are responsible for drafting the manuscript and revising it critically for important intellectual content, and have given final approval of the version to be submitted. CC, EC, FB, and SB conceived of the original study. All authors read and approved the final manuscript
